# Supplementary figures and images for: Mapping immune variation and var gene switching in naive hosts infected with Plasmodium falciparum
Source: eLife. 2021 Mar 2;10:e62800. doi: 10.7554/eLife.62800 (PMC7924948; doi:10.7554/eLife.62800)

inflammatory v016

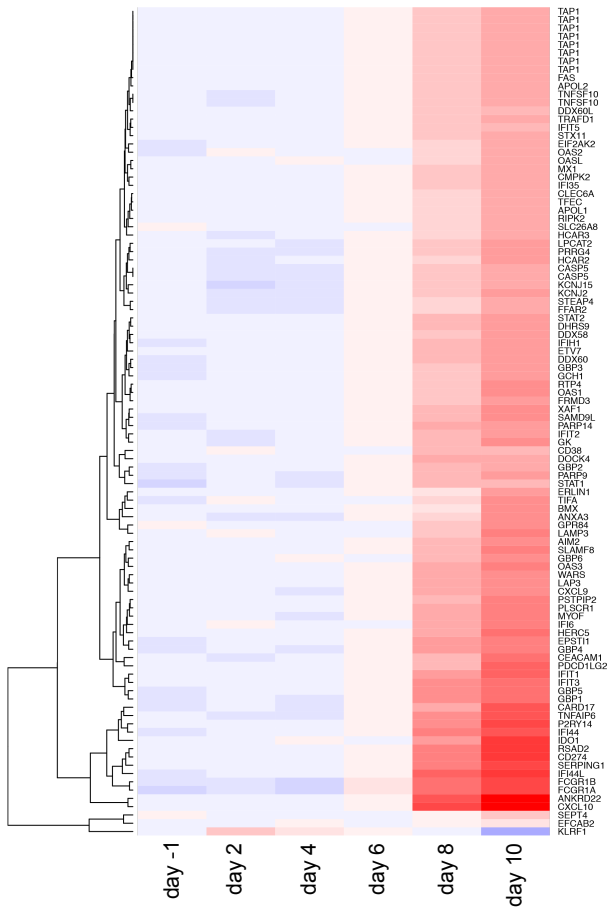

inflammatory v017

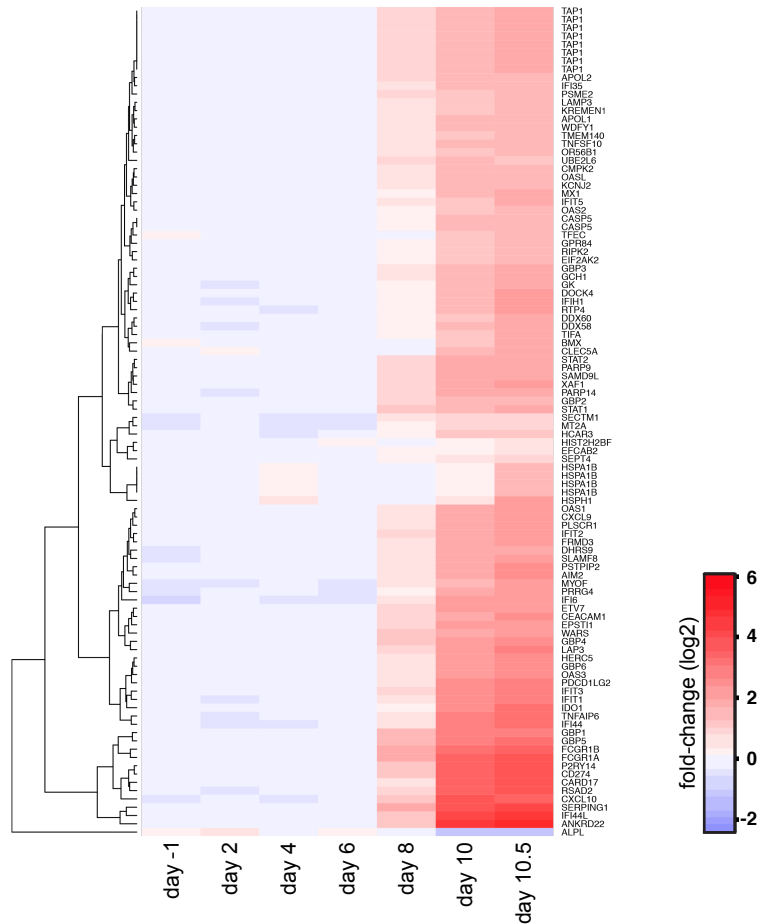

inflammatory v013

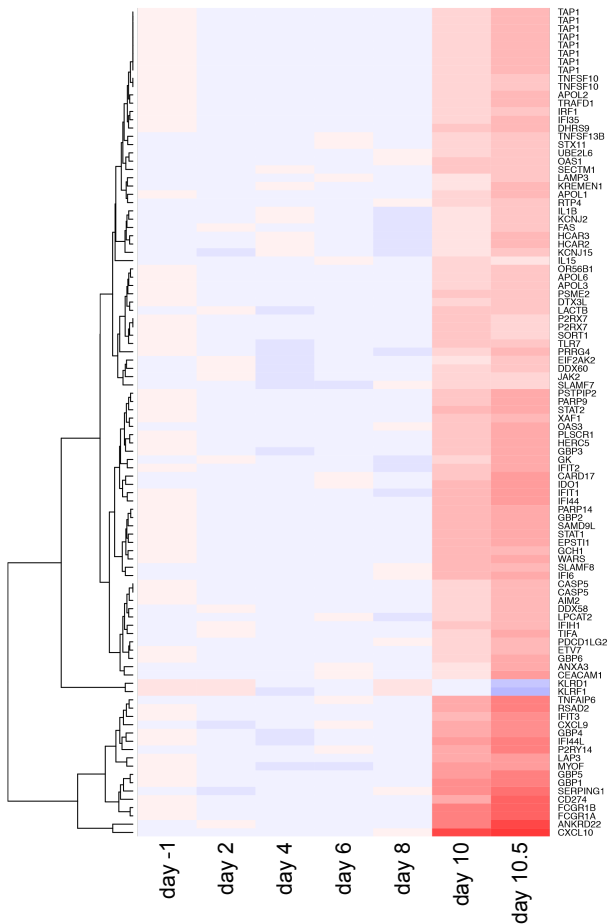

inflammatory v027

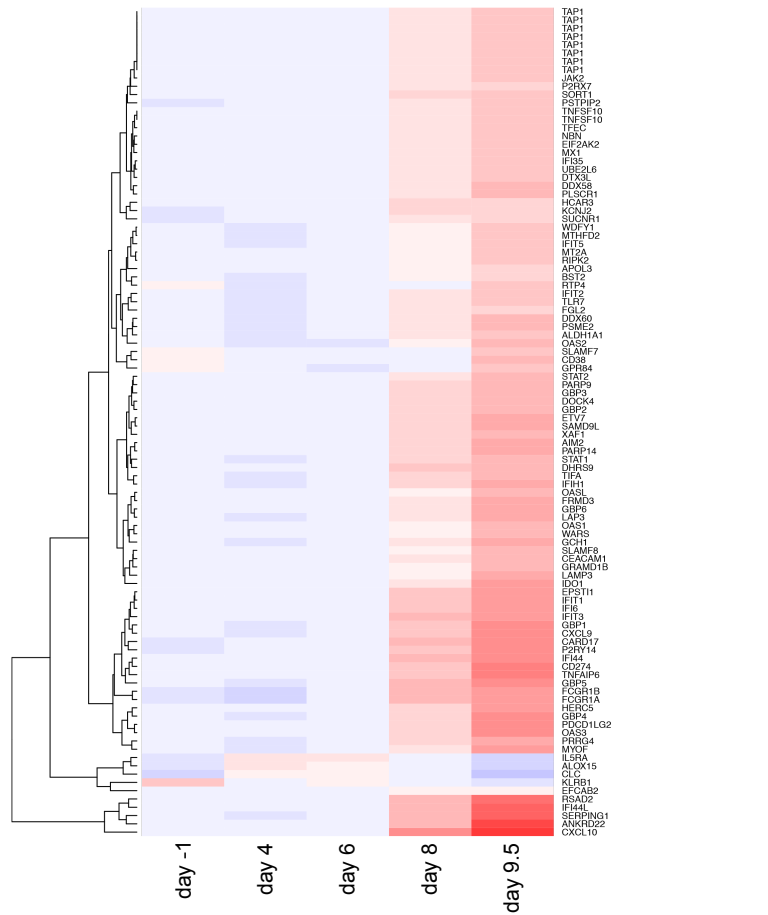



suppressor v022

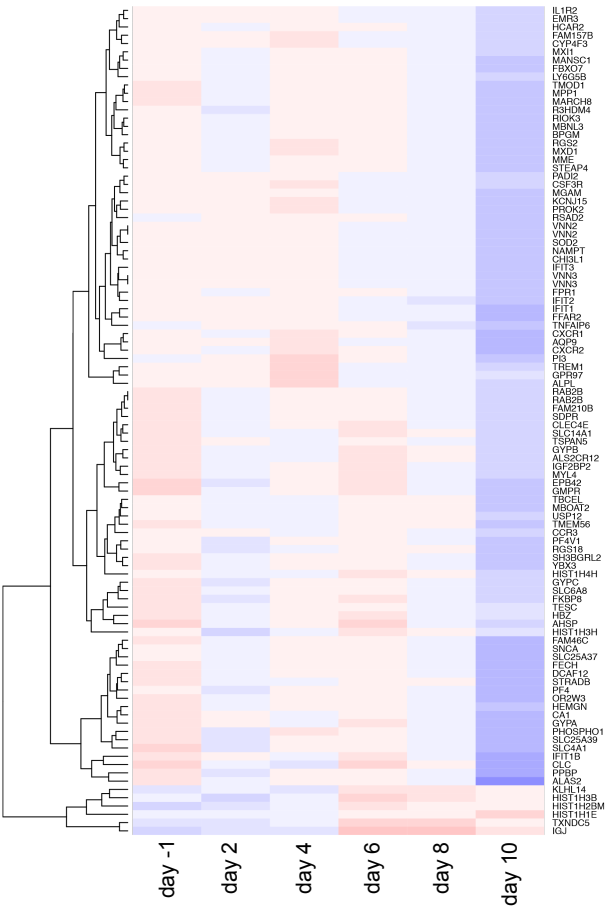

suppressor v019

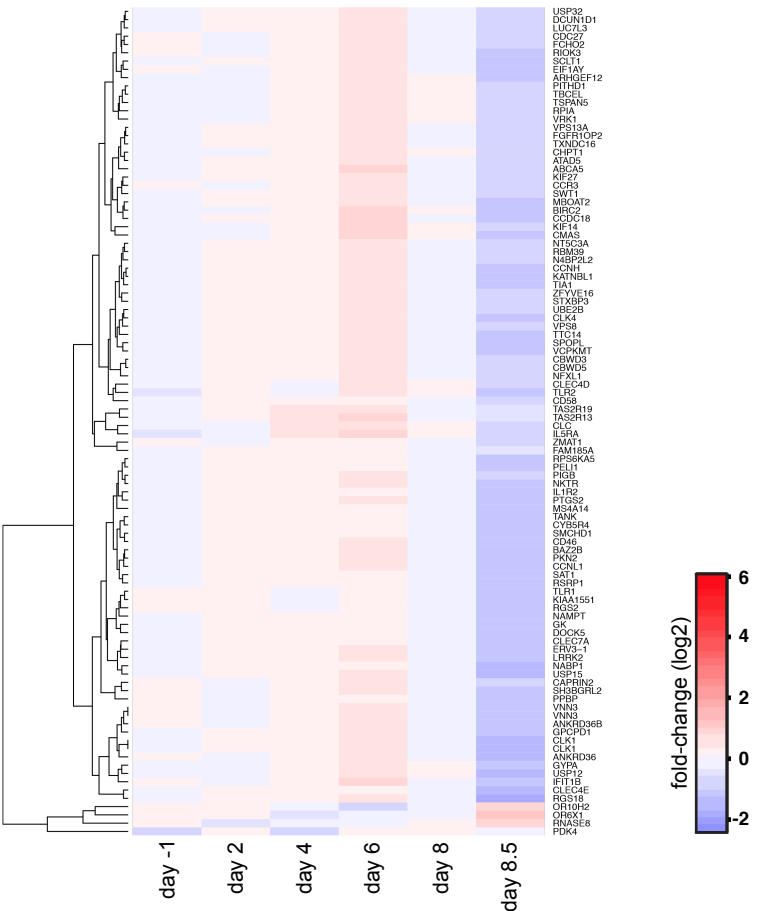

Supplement: Figure 1—source data 1. — Log2 fold-change of the 100 protein-coding genes with highest variance in whole blood during infection. Data are presented as deviation from median (within each volunteer). Genes (rows) are ordered by hierarchical clustering, whereas whole blood samples (columns) are ordered by time-point (pre-infection to diagnosis, left to right). Each volunteer was analysed independently and therefore every top 100 gene list is unique. [file elife-62800-fig1-data1.pdf]
