# Supplementary material for: Mapping immune variation and var gene switching in naive hosts infected with Plasmodium falciparum
Source: eLife. 2021 Mar 2;10:e62800. doi: 10.7554/eLife.62800 (PMC7924948; doi:10.7554/eLife.62800)
Supplement: Transparent reporting form [file elife-62800-transrepform.docx]

***eLife’s* transparent reporting form**

We encourage authors to provide detailed information *within their submission* to facilitate the interpretation and replication of experiments. Authors can upload supporting documentation to indicate the use of appropriate reporting guidelines for health-related research (see [EQUATOR Network](http://www.equator-network.org/%20)), life science research (see the [BioSharing Information Resource](https://biosharing.org/)), or the [ARRIVE guidelines](http://www.plosbiology.org/article/info:doi/10.1371/journal.pbio.1000412) for reporting work involving animal research. Where applicable, authors should refer to any relevant reporting standards documents in this form.

If you have any questions, please consult our Journal Policies and/or contact us: [editorial@elifesciences.org](mailto:editorial@elifesciences.org).

**Sample-size estimation**

- You should state whether an appropriate sample size was computed when the study was being designed
- You should state the statistical method of sample size computation and any required assumptions
- If no explicit power analysis was used, you should describe how you decided what sample (replicate) size (number) to use

Please outline where this information can be found within the submission (e.g., sections or figure legends), or explain why this information doesn’t apply to your submission:

This study presents the results of immunological and parasitological analyses of healthy human volunteers enrolled as controls (i.e. non-vaccinated) in a Phase I/IIa efficacy trial of the FMP2.1/AS01B malaria vaccine. The power calculations used to inform trial design have been published previously (Payne *et al*, 2016, PMID: 26908756) but are copied below for ease of access:

Prior to undertaking the study, power calculations were performed by the Centre for Statistics in Medicine at the University of Oxford. Data were available from small studies undertaken with the same inoculum in Oxford, as well as at the Radboud University Nijmegen Medical Centre in the Netherlands. These historical data suggested the coefficient of variation (CV) in the controls may range from 22% (Nijmegen where mean PMR = 10) to 33% (Oxford where mean PMR = 12). A study design (where the maximum number of volunteers = 30) with 15 controls versus 15 vaccinees consistently provided the best power to observe a 33% reduction in mean PMR when allowing for this CV in the controls, and an increased CV in the vaccinees.

**Replicates**

- You should report how often each experiment was performed
- You should include a definition of biological versus technical replication
- The data obtained should be provided and sufficient information should be provided to indicate the number of independent biological and/or technical replicates
- If you encountered any outliers, you should describe how these were handled
- Criteria for exclusion/inclusion of data should be clearly stated
- High-throughput sequence data should be uploaded before submission, with a private link for reviewers provided (these are available from both GEO and ArrayExpress)

Please outline where this information can be found within the submission (e.g., sections or figure legends), or explain why this information doesn’t apply to your submission:

Two independent clinical trials (VAC054 and VAC065) were undertaken to examine the host response to falciparum malaria, and the switching and selection of parasite variant surface antigens. Details of each trial are provided in the Methods (see 'Study participants and ethical approval' and 'Controlled human malaria infection' and 'Monitoring volunteers') and in Supplementary File 1. Furthermore, direct links to ClinicalTrials.gov are provided in the Methods for each trial - this allows the reader to look up all details relating to trial design.

To quantify parasite gene expression in the inoculum used for blood challenge (VAC054) two technical replicates of a single biological sample were analysed. These were handled independently all the way from RNA extraction and clean-up to library preparation and barcoding to sequencing and data analysis. Details are provided in the Methods (see 'Isolation of parasites for *ex vivo* RNA-sequencing') and Figure Legends.

To analyse the response of each volunteer independently time-course analyses of parasite densities, symptomatology, plasma proteins and whole blood RNA were measured through time. Power was therefore provided by performing repeat measurements. Details of the number of samples/time-points are provided in the Methods and the Figure Legends. As we were interested in identifying individuals whose response could be considered an "outlier" (this was a key objective of the study) no data were excluded during analysis. However, it was not always possible to successfully generate data for every volunteer (e.g. parasite RNA-sequencing) and in all of these cases, we specify the volunteers for whom data was successfully generated and details are provided throughout the Results, Methods and Figure Legends.

All RNA-seq / microarray datasets are published and can be accessed here:

<https://www.ncbi.nlm.nih.gov/geo/query/acc.cgi?acc=GSE132050>

<https://www.ebi.ac.uk/ena/browser/view/ERP116360>

<https://www.ebi.ac.uk/ega/studies/EGAS00001003766>

**Statistical reporting**

- Statistical analysis methods should be described and justified
- Raw data should be presented in figures whenever informative to do so (typically when N per group is less than 10)
- For each experiment, you should identify the statistical tests used, exact values of N, definitions of center, methods of multiple test correction, and dispersion and precision measures (e.g., mean, median, SD, SEM, confidence intervals; and, for the major substantive results, a measure of effect size (e.g., Pearson's r, Cohen's d)
- Report exact p-values wherever possible alongside the summary statistics and 95% confidence intervals. These should be reported for all key questions and not only when the p-value is less than 0.05.

Please outline where this information can be found within the submission (e.g., sections or figure legends), or explain why this information doesn’t apply to your submission:

All statistical tests carried out to support the conclusions of this study (whether significant or not significant) are detailed throughout the Results, Figure Legends and Methods. As the objective of this study was to analyse the response of each volunteer independently we show the data for each individual volunteer and sample/time-point in all Figures. The raw data that underpin heatmaps (microarray and RNA-sequencing) are included as source data for Figures 1 and 4, respectively.

Details of the methodology used to call differentially expressed genes (microarray and RNA-sequencing) and differentially abundant metabolites (metabolomics) are provided in the Methods and Results. We also specify the sample number (N) for each pairwise comparison, the method used to adjust for multiple testing and the adjusted p value that we considered significant. Many of these details are also given in the Figure Legends, where appropriate. Note that we also report on differential gene expression analyses that yielded no significant hits and these are described in the Results. Summary statistics of the microarray dataset are provided in Supplementary File 2 and details of these stats are given in the legend.

For all other data types (e.g. symptomatology, parasite multiplication rate, blood counts etc.) we provide the statistical method used to assess significance, the number of volunteers and/or datapoints, the exact p value, and the 95% confidence intervals (where appropriate). We also include information on median and range when we feel that this additonal information will help the reader assess the significance of a finding. All of these details are given throughout the Results, Figure Legends and Methods.

(For large datasets, or papers with a very large number of statistical tests, you may upload a single table file with tests, Ns, etc., with reference to sections in the manuscript.)

**Group allocation**

- Indicate how samples were allocated into experimental groups (in the case of clinical studies, please specify allocation to treatment method); if randomization was used, please also state if restricted randomization was applied
- Indicate if masking was used during group allocation, data collection and/or data analysis

Please outline where this information can be found within the submission (e.g., sections or figure legends), or explain why this information doesn’t apply to your submission:

Allocation to study group (vaccinee or control) was based on time of enrolment (vaccinees were enrolled before controls) and volunteer preference; randomisation was not included in the study design. Note that all clinical staff were blinded to which group the volunteers were assigned. Further details of group allocation and blinding can be found here:

https://clinicaltrials.gov/ct2/show/NCT02044198?term=NCT02044198&draw=2&rank=1

Only control volunteers were analysed in this study.

**Additional data files (“source data”)**

- We encourage you to upload relevant additional data files, such as numerical data that are represented as a graph in a figure, or as a summary table
- Where provided, these should be in the most useful format, and they can be uploaded as “Source data” files linked to a main figure or table
- Include model definition files including the full list of parameters used
- Include code used for data analysis (e.g., R, MatLab)
- Avoid stating that data files are “available upon request”

Please indicate the figures or tables for which source data files have been provided:

Source Data and Supplementary Files

Supplementary File 1	volunteer demographics for VAC054 and VAC065

Supplementary File 2	supports Figure 1 - supplement 1 & 2

Supplementary File 3	supports Figure 2

Supplementary File 4	supports Figure 2

Supplementary File 5	supports Figure 2

Supplementary File 6	supports Figure 1 and Figure 2

Supplementary File 7	supports Figure 2 - supplement 1

Supplementary File 8	supports Figure 2 - supplement 1

Figure 1 - source data 1	raw data that underpins the heatmap in Figure 1

Figure 4 - source data 1	raw data that underpins the heatmap in Figure 4
